# Geographic Validation of the SADFUL Scores for Identifying Bacteremia in the Unscheduled Emergency Department Revisit Cohorts

**DOI:** 10.1155/emmi/1195292

**Published:** 2026-06-16

**Authors:** Cheng-Yi Fan, Chun-Ju Lien, Po-Lin Wang, Chi-Hsin Chen, Jiun-Wei Chen, Edward Pei-Chuan Huang, Chih-Wei Sung

**Affiliations:** ^1^ Department of Emergency Medicine, National Taiwan University Hospital Hsin-Chu Branch, Hsinchu, Taiwan, hch.gov.tw; ^2^ Institute of Molecular Medicine, National Tsing Hua University, Hsinchu, Taiwan, nthu.edu.tw; ^3^ Department of Emergency Medicine, College of Medicine, National Taiwan University, Taipei, Taiwan, ntu.edu.tw; ^4^ Department of Family Medicine, National Taiwan University Hospital Hsin-Chu Branch, Hsinchu, Taiwan, hch.gov.tw; ^5^ Department of Emergency Medicine, National Taiwan University Hospital, Taipei, Taiwan, ntuh.gov.tw

**Keywords:** bacteremia, emergency department, revisit, SADFUL score, validation

## Abstract

**Background:**

The SADFUL score is developed to identify bacteremia in emergency department revisits (EDR), but its generalizability in various healthcare facilities and superiority over other scores remain unclear. This study aims to validate the SADFUL score in two hospitals with different levels of care.

**Method:**

This retrospective cohort study included adult EDR patients from an academic medical center (Hospital A) and a rural regional hospital (Hospital B) between March 1, 2019, and December 31, 2022. The SADFUL, quick sequential organ failure assessment (qSOFA), systemic inflammatory response syndrome (SIRS) criteria, and shock index (SI) scores were compared using the area under the receiver operating characteristic curve (AUROC).

**Results:**

The prevalence of bacteremia in the EDR cohort was 2.2% (120/5455). The SADFUL score’s performance was inconsistent across the two hospitals. In Hospital A, its AUROC of 0.64 was significantly higher than those of the SIRS criteria, qSOFA, and the SI. In contrast, in Hospital B, its numerically higher AUROC of 0.72 was not statistically superior to the other scores. The optimal cutoff also differed between the two settings (≥ 2 in Hospital A and ≥ 4 in Hospital B).

**Conclusion:**

The SADFUL score demonstrated only modest accuracy and was not consistently superior to other commonly used clinical scoring systems. Overall, all evaluated tools exhibited limited utility in identifying bacteremia among EDR patients, highlighting the need for further research in this area.

## 1. Background

The early detection of bacteremia is a critical decision in the emergency department (ED), as delayed recognition may lead to sepsis progression, while unnecessary workup contributes to antibiotic overuse. The decision is particularly challenging in patients with unscheduled emergency department revisits (EDR) within 72 h, who often present with a worsening condition or an overlooked diagnosis from their initial visit [1]. This scenario introduces significant diagnostic uncertainty, making it difficult for clinicians to differentiate potentially life‐threatening conditions such as bacteremia from more benign causes.

To predict bacteremia, various models and scoring systems have been developed to help in decision‐making on whether to conduct blood cultures and initiate antibiotic therapy. These tools range from general severity scores, such as the systemic inflammatory response syndrome (SIRS) criteria, quick sequential organ failure assessment (qSOFA), shock index (SI), and modified early warning score (MEWS), to models specifically designed to predict bacteremia itself [2–4]. The Shapiro rule, which uses clinical data, vital signs, chief complaints, and blood test results, has a high sensitivity at 94%–97% but a low specificity at 28%–48% [5, 6]. Lee et al. used a higher threshold for body temperature, thereby achieving a balance between sensitivity (83.3%) and specificity (82.4%) [7]. A comprehensive meta‐analysis revealed that the area under the receiver operating characteristic curve (AUROC) for procalcitonin in predicting bacteremia is 0.84 (95% CI 0.75–0.90) [8]. Other predictive models using a combination of biomarkers, such as C‐reactive protein and procalcitonin, and other common variables are associated with improved efficacy, with AUROCs of 0.85–0.95 [9–11]. However, none of these scores were particularly focused on bacteremia in EDR.

Our previous research introduced the SADFUL scoring system, which is specifically designed to identify bacteremia in patients with EDR [12]. This model incorporated various factors, including a segmented neutrophil percentage of > 85%, age over 55 years, diabetes mellitus, fever, upper respiratory tract symptoms, and leukopenia. In our cohort, the SADFUL scoring system had a moderate discriminative power, with an AUROC of 0.79 (95% CI 0.71–0.88). Notably, it surpassed the performance of the qSOFA, SIRS, and SI in bacteremia association. However, the development and internal validation of the SADFUL score were performed at a single regional hospital, causing uncertainty on its generalization across different healthcare settings. Therefore, this study aimed to conduct an external validation of the SADFUL score in patients with EDR at two distinct healthcare facilities.

## 2. Method

### 2.1. Study Design and Cohort

This retrospective cohort study performed an external validation of the SADFUL scoring system using data from Hospital A (an academic tertiary medical center in Taipei City, Taiwan) and Hospital B (a secondary regional hospital located in Taiwan’s largest agricultural county). The average monthly patient visits to the EDs of Hospitals A and B were 9000 and 4000, respectively, as shown in Supporting Table 1. Patients who revisited the ED within 72 h after being discharged in the index visit were included in the EDR cohort. Then, to identify patients with potential infectious disease, we selected those who underwent examination of complete blood cell count (CBC) and differential count (DC) of white blood cells (WBCs) and two sets of blood cultures from March 1, 2019, to December 31, 2022. The exclusion criteria included patients aged under 18 years, those discharged against medical advice (DAMA), and those with incomplete data on the requisite components of the SADFUL scores. In our institutional electronic medical record system, certain charts are access‐restricted when they involve potential legal proceedings, rendering the triage records, chief complaints, and present illness unretrievable. These patients were excluded because two SADFUL components—upper respiratory tract symptoms and body temperature—could not be ascertained.

This study was approved by the Institutional Review Board, and informed consent was waived. This study was performed in accordance with the Strengthening the Reporting of Observational Studies in Epidemiology (STROBE) guidelines [13] (Supporting File: STROBE_checklist_R2_2). No patients or public were involved in the design of the study.

### 2.2. Data Source, Processing, and Management

This study was conducted as a retrospective chart review, adhering to methodological standards as outlined by Worster and Bledsoe [14]. Data were collected in two phases. In the first phase, structured data including age, sex, triage level, vital signs, Glasgow Coma Scale score, laboratory test results, and disposition decisions were extracted from an integrated medical database. The triage level was based on the Taiwan Triage and Acuity Scale, which prioritizes the patients by five levels [15].

In the second phase, six emergency physicians served as trained abstractors to identify preexisting conditions including hypertension, diabetes mellitus, coronary artery disease, cerebrovascular accidents, chronic kidney disease, and cancer. In addition, they recorded the five primary complaints presented during the ED visits, including upper respiratory tract infections, gastrointestinal disorders, urinary tract infections, dyspnea, and chills. Any discrepancies or uncertainties were collaboratively resolved via a discussion among the team members. Prior to abstraction, all six physicians received standardized training on case selection criteria and variable definitions and were provided a prespecified abstraction form. Their performance was monitored after the first 20 charts, and all abstractors were blinded to the study hypothesis. The data abstraction process followed the same rigorous methodology as our group’s previous work using this database. The reliability of this established process was high, with a previously reported kappa statistic of 0.87 and an intraclass correlation coefficient of 0.93 [16].

### 2.3. Definition of Bacteremia

Bacteremia was defined in accordance with the guidelines of the Centers for Disease Control and Prevention. A true case of bacteremia was identified using one or more of the following criteria [17]: (1) positive results from two or more sets of blood culture drawn from different sites or (2) a single set of positive gram‐negative blood culture. Conversely, a single set of gram‐positive blood cultures commonly indicated contamination or colonization rather than active infection. Samples were processed using the BACTEC 9240 system (Becton Dickinson and Company, BD). Blood samples were incubated for up to 5 days or until bacterial growth was detected.

### 2.4. Clinical Decision Rules

In this study, the primary outcome was bacteremia, and the SADFUL score was calculated at the revisit for each participant in the validation cohort. The simplified score was calculated as follows: “S” (segmented neutrophil percentage of > 85%, +3 points), “A” (age > 55 years, +1 point), “D” (diabetes mellitus, +1 point), “F” (fever, body temperature ≥ 38°C, +2 points), “U” (upper respiratory tract symptoms, −2 points), and “L” (leukopenia, WBC count < 4 K/μL, +2 points). Other sepsis‐predicting scores and indices were also calculated and compared with the SADFUL score using the following criteria: (1) qSOFA, with a positive result defined as meeting ≥ 2 criteria, including a systolic blood pressure of ≤ 100 mmHg, respiratory rate of ≥ 22 cycles/min, and altered mental status (Glasgow Coma Scale score of < 15); (2) SIRS criteria, with a positive result defined as meeting ≥ 2 criteria, including heart rate of > 90 beats/min, respiratory rate of > 20 cycles/min, body temperature of < 36°C or ≥ 38°C, and WBC count of > 12 or < 4 K/μL; and (3) SI, with a positive result defined as heart rate/systolic blood pressure of ≥ 1. The clinical rules are summarized in Supporting Table 2.

### 2.5. Statistical Analysis

In the descriptive analysis, differences between patients with bacteremia and those without bacteremia were evaluated using the independent *t* test for continuous variables, chi‐square test for dichotomous variables, and Mann–Whitney *U* test for nonparametric variables. Multiple logistic regression analyses were used to identify the factors associated with bacteremia. For the external validation, the SADFUL, qSOFA, and SIRS criteria scores and SI of each participant in the validation cohort were calculated. Patients with any missing values in the components of these rules were excluded from the analysis. Then, the prevalence of bacteremia across the different models was assessed. Key statistical measures, including the AUROC, sensitivity, specificity, positive predictive value (PPV), negative predictive value (NPV), positive likelihood ratio (PLR), negative likelihood ratio (NLR), and accuracy, were calculated. The DeLong test was used to compare the AUROC across different scores. All statistical analyses were conducted using the Statistical Package for the Social Sciences software Version 26.0 (Armonk, NY: IBM Corporation Inc.), and a *p* value of 0.05 was considered a statistically significant difference.

To evaluate the concordance between the predicted probabilities and the actual outcomes, calibration curve analysis was performed. The analysis involved the six variables of the SADFUL score, and it was conducted using the calibration curves package in the R software [18].

### 2.6. Sensitivity Analysis: Propensity Score Matching

A sensitivity analysis using propensity score matching (PSM) was conducted to account for potential selection bias and confounding factors. The propensity score was calculated for each patient via a multivariable logistic regression model that included 10 covariates representing two domains: baseline comorbidities associated with bacteremia risk and the clinical decision to order blood cultures (sex, hypertension, diabetes mellitus, coronary artery disease, cancer, and chronic kidney disease), and acute clinical presentations reflecting illness severity at the index revisit (chills, triage level 1 or 2, respiratory rate > 20/min, and systolic blood pressure < 100 mmHg). These variables were selected as potential confounders on clinical grounds, and notably do not overlap substantially with the SADFUL score components, with the exception of diabetes mellitus, which contributes only 1 of 9 possible points in the score. Each patient with bacteremia was then matched to four patients without bacteremia (1:4 ratio) using a nearest‐neighbor algorithm with a caliper width of 0.2 of the standard deviation of the logit of the propensity score. The balance of covariates was assessed using standardized mean differences (SMD < 0.1), after which the AUROCs for all clinical scores were recalculated in the matched cohort.

## 3. Results

### 3.1. Demographic Characteristics of the Participants

**FIGURE 1:**
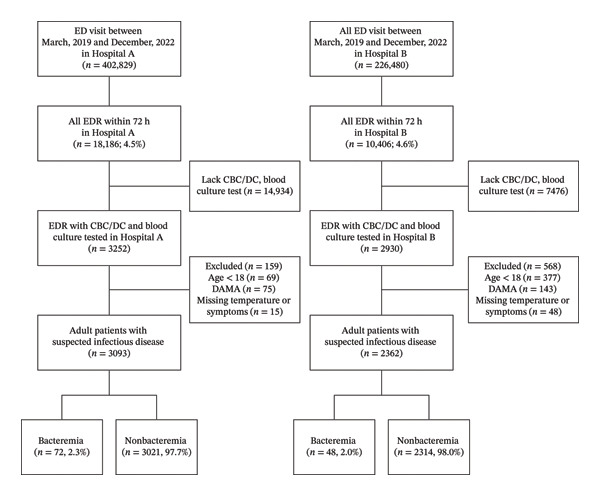
The inclusion and exclusion processes for retrospective review of emergency department revisits for suspected infection and finally diagnosed with bacteremia or not in two hospitals in Taiwan.

Figure 1 shows the patient enrollment process. The EDR rates in the two hospitals were 4.5% and 4.6%, respectively. In Hospital A, 3093 of 18,186 patients with EDRs were eligible for analysis, and 72 (2.3%) patients were diagnosed with true bacteremia. In Hospital B, 2362 of 10,406 patients with EDRs were eligible for analysis, and 48 (2.0%) patients were diagnosed with true bacteremia.

Table 1 presents the demographic characteristics and clinical findings for patients in Hospitals A and B. In Hospital A, several differences in baseline characteristics were observed. Patients with bacteremia tended to be older (mean age: 65.6 vs. 60.5 years, *p* = 0.027) and showed a higher prevalence of hypertension (56.9% vs. 35.8%, *p* < 0.001), coronary artery disease (23.6% vs. 13.7%, *p* = 0.016), and chronic kidney disease (30.6% vs. 13.7%, *p* < 0.001) than those without bacteremia. Moreover, patients with bacteremia had higher hospital admission rates (86.1% vs. 57.9%, *p* < 0.001) and a longer length of hospital stay (median: 18.0 vs. 10.0 days, *p* < 0.001). Other characteristics, such as chief complaints, triage levels, and vital signs, did not show notable differences between groups.

**TABLE 1:** Baseline characteristics of bacteremia and nonbacteremia patients in hospital A.

	**Hospital A**				**Hospital B**	
**Dataset**	**Bacteremia (*n* = 72), *n* (%)**	**Nonbacteremia (*n* = 3021), *n*(%)**	**p** **value**	**Bacteremia (*n* = 48), *n* (%)**	**Nonbacteremia (*n* = 2314), *n* (%)**	**p** **value**

Age (years)	65.6 ± 19.1	60.5 ± 19.0	0.027	69.6 ± 16.2	62.8 ± 19.5	0.007
> 55 years	53 (73.6)	1952 (64.6)	0.114	40 (83.3)	1544 (66.7)	0.015
Males	39 (54.2)	1550 (51.3)	0.631	29 (60.4)	1282 (55.4)	0.489
Preexisting diseases						
Hypertension	41 (56.9)	1083 (35.8)	< 0.001	21 (43.8)	1066 (46.1)	0.750
DM	20 (27.8)	645 (21.4)	0.190	19 (39.6)	735 (31.8)	0.250
CAD	17 (23.6)	413 (13.7)	0.016	7 (14.6)	217 (9.4)	0.223
CVA	5 (6.9)	207 (6.9)	1.000	4 (8.3)	173 (7.5)	0.823
Cancer	28 (38.9)	1103 (36.5)	0.679	13 (27.1)	483 (20.9)	0.296
CKD	22 (30.6)	413 (13.7)	< 0.001	12 (25.0)	338 (14.6)	0.045
Chief complaints						
Upper respiratory tract	8 (11.1)	519 (17.2)	0.176	11 (22.9)	563 (24.3)	0.821
Gastrointestinal tract	30 (41.7)	1402 (46.4)	0.425	26 (54.2)	1249 (54.0)	0.979
Urinary tract	7 (9.7)	435 (14.4)	0.262	3 (6.3)	315 (13.6)	0.139
Dyspnea	10 (13.9)	549 (18.2)	0.351	12 (25.0)	439 (19.0)	0.293
Chills	20 (27.8)	573 (19.0)	0.061	8 (16.7)	188 (8.1)	0.034
Triage level 1 or 2	19 (26.4)	663 (21.9)	0.369	19 (39.6)	426 (18.4)	< 0.001
Vital signs						
GCS < 15	6 (8.3)	198 (6.6)	0.548	7 (14.6)	222 (9.6)	0.248
Body temperature			0.011			< 0.001
< 37.5°C	31 (43.1)	1776 (58.8)		19 (39.6)	1515 (65.5)	
≥ 37.5°C	41 (56.9)	1245 (41.2)		29 (60.4)	799 (34.5)	
Heart rate > 90 bpm	44 (61.1)	1856 (61.4)	0.955	33 (68.8)	1340 (57.9)	0.132
Breath rate > 20 times/min	11 (15.3)	571 (18.9)	0.437	10 (20.8)	118 (5.1)	< 0.001
SBP < 100 mmHg	12 (16.7)	341 (11.3)	0.156	9 (18.8)	132 (5.7)	< 0.001
Laboratory test						
WBC			0.137			0.024
< 4 K/μL	7 (9.7)	227 (7.5)		6 (12.5)	130 (5.6)	
> 12 K/μL	24 (33.3)	739 (24.5)		18 (37.5)	642 (27.7)	
Segmented neutrophils (%)	79.2 ± 16.7	75.8 ± 14.2	0.088	79.1 ± 19.0	75.9 ± 13.1	0.258
Admission	62 (86.1)	1749 (57.9)	< 0.001	40 (83.3)	1426 (61.6)	0.002
Length of stays	18.0 [11.0, 33.0]	10.0 [6.0,17.0]	< 0.001	13.5 [7.0, 25.8]	8.0 [5.0, 14.0]	< 0.001

*Note:* Data are presented as number (%).

Abbreviations: CAD, coronary artery disease; CKD, chronic kidney disease; CT, computed tomography; CVA, cerebrovascular accident; DM, diabetes mellitus; GCS, Glasgow Coma Scale; SBP, systolic blood pressure; WBC, white blood cell.

In Hospital B, patients with bacteremia were also older (average age: 69.6 vs. 62.8 years, *p* = 0.007) and presented with more clinical signs, including higher triage levels (39.6% vs. 18.4% at level 1 or 2, *p* < 0.001), higher respiratory rates (> 20 cycles/min, 20.8% vs. 5.1%, *p* < 0.001), higher body temperatures (> 38°C, 52.1% vs. 21.1%, *p* < 0.001), and a greater likelihood of having a low systolic blood pressure (< 100 mmHg, 18.8% vs. 5.7%, *p* < 0.001). Similar to Hospital A, patients with bacteremia in Hospital B also had higher hospital admission rates (83.3% vs. 61.6%, *p* = 0.002) and a longer length of hospital stay (median: 13.5 vs. 8.0 days, *p* < 0.001).

In terms of the pathogens identified, *Staphylococcus aureus* and *Pseudomonas aeruginosa* were the most common pathogens, followed by Salmonella O9 and *Staphylococcus epidermidis* (Supporting Table 3). The contamination rates in blood cultures were 3.0% in Hospital A and 3.6% in Hospital B.

### 3.2. Factors Associated With Bacteremia

The results of the multiple logistic regression analysis to identify factors independently associated with bacteremia are presented in Supporting Table 4. In Hospital A, after adjusting for other variables, factors associated with an increased odds of bacteremia were a history of hypertension (aOR 1.75, 95% CI 1.02–3.00), chronic kidney disease (aOR 2.19, 95% CI 1.28–3.77), and a body temperature > 38°C (aOR 1.83, 95% CI 1.07–3.12).

In Hospital B, the independent predictors were a body temperature > 38°C (aOR 3.87, 95% CI 2.04–7.32), a respiratory rate > 20/min (aOR 3.41, 95% CI 1.47–7.91), a systolic blood pressure < 100 mmHg (aOR 3.59, 95% CI 1.54–8.34), and a white blood cell count < 4 K/μL (aOR 2.70, 95% CI 1.04–7.03).

### 3.3. Performance of the SADFUL Score

**FIGURE 2:**
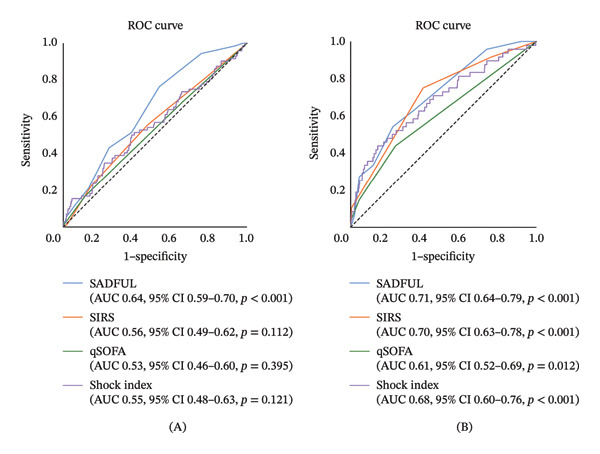
The receiver operating characteristic curve of the SADFUL, SIRS, qSOFA, and SI scores for identifying bacteremia in (A) Hospital A and (B) Hospital B.

The performance metrics of the SADFUL score at various cutoff points in Hospital A are detailed in Table 2. Across the tested thresholds of ≥ 2, ≥ 3, and ≥ 4, the sensitivity ranged from 0.43 to 0.77 and the specificity from 0.48 to 0.75. The PLR ranged from 1.38 to 1.72, while the NLR ranged from 0.50 to 0.77. The NPVs were consistently 0.98–0.99. A cutoff of ≥ 2 yielded the highest sensitivity (0.77), whereas a cutoff of ≥ 4 resulted in the highest accuracy (0.74). The overall AUROC for the SADFUL score was 0.64. This value was significantly higher than the AUROCs for the SIRS criteria (0.56), qSOFA (0.53), and the SI (0.56), with DeLong test *p* values of 0.018, 0.003, and 0.027, respectively (Figure 2A).

**TABLE 2:** Performance of the SADFUL score with different cutoffs.

	**SADFUL**	**Sensitivity**	**Specificity**	**PPV**	**NPV**	**PLR**	**NLR**	**DOR**	**Accuracy**

Hospital A	≥ 2	0.76	0.48	0.03	0.99	1.46	0.50	2.95	0.48
	≥ 3	0.51	0.63	0.03	0.98	1.38	0.77	1.79	0.63
	≥ 4	0.43	0.75	0.04	0.98	1.72	0.76	2.27	0.74

Hospital B	≥ 2	0.77	0.49	0.03	0.99	1.51	0.47	3.24	0.50
	≥ 3	0.63	0.66	0.04	0.99	1.86	0.56	3.30	0.66
	≥ 4	0.54	0.77	0.05	0.99	2.37	0.59	4.00	0.77

Abbreviations: DOR, diagnostic odds ratio; NLR, negative likelihood ratio; NPV, negative predictive value; PLR, positive likelihood ratio; PPV, positive predictive value.

In Hospital B, the performance metrics for the SADFUL score are also presented in Table 2. At the tested cutoffs of ≥ 2, ≥ 3, and ≥ 4, sensitivity ranged from 0.54 to 0.77, and specificity ranged from 0.49 to 0.77. The PLR was between 1.51 and 2.37, while the NLR was between 0.47 and 0.59. The NPVs were consistently 0.99. A cutoff of ≥ 2 provided the highest sensitivity (0.77), while the cutoff of ≥ 4 resulted in the highest accuracy (0.77). The overall AUROC for the SADFUL score was 0.72. However, its performance was not statistically superior to that of the SIRS criteria (0.70), qSOFA (0.61), or the SI (0.68), as determined by the DeLong test (*p* > 0.05 for all comparisons) (Figure 2B).

**FIGURE 3:**
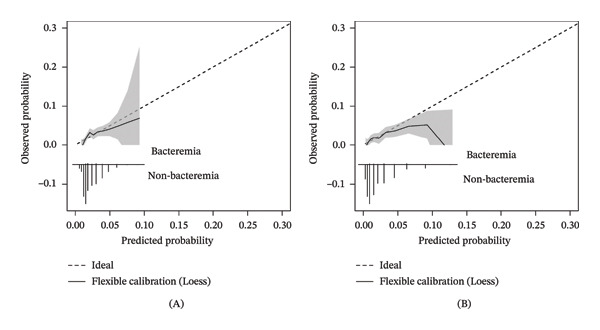
The flexible calibration curve using the SADFUL score for identifying bacteremia in (A) Hospital A and (B) Hospital B.

Figure 3 shows the flexible calibration curves for both cohorts. These curves, derived from the logistic regression model, were closely associated with the ideal diagonal line at lower predicted probabilities in both hospitals. Most samples had a predicted probability below 0.25 due to the low true bacteremia rate. However, the calibration curves indicated an overestimation of risk at higher predicted probabilities.

### 3.4. Sensitivity Analysis With Propensity Score Matching

After 1:4 PSM, the sensitivity analysis cohort consisted of 120 patients with bacteremia and 480 patients without bacteremia. The baseline characteristics between these two populations are shown in Supporting Table 5. The overall discriminative performance of the SADFUL score in this matched cohort is presented in the receiver operating characteristic curve (Supporting Figure 1). The AUROC was 0.62 (95% CI 0.57–0.68, *p* < 0.001). The detailed performance metrics at different cutoffs are shown in Supporting Table 6. At the tested thresholds of ≥ 2, ≥ 3, and ≥ 4, the sensitivity ranged from 0.48 to 0.77 and the specificity from 0.43 to 0.69. The PLR ranged from 1.33 to 1.52, while the NLR ranged from 0.55 to 0.76. The calibration of the SADFUL score is illustrated in Supporting Figure 2.

## 4. Discussion

This external validation of the SADFUL score across two distinct hospital settings reveals two primary findings. First, the score demonstrated only limited to modest discriminative capacity, with AUROCs lower than in the original derivation study. Second, its performance relative to other clinical scores was inconsistent; while it showed a statistically significant advantage in the academic medical center (Hospital A), this superiority was not observed in the regional hospital (Hospital B). These findings suggest that the utility and generalizability of the SADFUL score require further validation.

A critical finding of this study is the variation in risk score performance between two hospital settings. Given that Hospital A shares a similar tertiary‐care infrastructure with the original derivation setting, its performance aligns with previous expectations. In contrast, the findings from the rural regional hospital (Hospital B) are characterized by a more pronounced clinical manifestation—a “high‐severity phenotype”—that suggests a distinct disease trajectory. In Hospital B, all evaluated scoring systems, including SIRS, qSOFA, and the SI, demonstrated substantially higher AUROCs compared to Hospital A. This consistent trend suggests that the performance difference is rooted in fundamental variations in patient cohorts. In Hospital B, the strongest predictors were acute clinical signs, such as fever (aOR 3.87), tachypnea (aOR 3.41), hypotension (aOR 3.59), and leukopenia (aOR 2.70), whereas Hospital A’s predictors were primarily underlying chronic comorbidities. This divergence may be partly explained by differences in disease awareness: patients in rural settings are less likely to have documented or self‐reported comorbidities, which could attenuate the predictive contribution of chronic disease variables in that context.

The predominance of this acute clinical presentation likely explains why a higher cutoff score (≥ 4) was optimal in Hospital B. However, the observation that these patients were initially discharged during their index visit points to the dynamic evolution of bacteremia. In its earliest stages, the clinical presentation may be nonspecific or lack the “red flags” that typically trigger admission. Furthermore, rural healthcare‐seeking behaviors often involve delays in seeking care due to limited accessibility. Patients in rural regions may delay seeking care, and therefore present at a more advanced and clinically apparent stage of illness [19]. This hypothesis is supported by our triage data, in which patients with bacteremia in Hospital B were twice as likely to be assigned a high‐acuity triage level (Level 1 or 2) compared to those in Hospital A. Thus, the increased discriminative power of traditional vital signs in Hospital B reflects a more advanced disease stage at the point of the unscheduled revisit.

These observations collectively suggest that the discriminative performance and optimal threshold of the SADFUL score are meaningfully shaped by local case mix and institutional context. Beyond differences in disease severity at presentation, factors such as healthcare‐seeking behavior, referral patterns, and variations in clinical practice between a tertiary medical center and a regional hospital may all contribute to this heterogeneity. Taken together, these findings imply that SADFUL may not be fully transportable across settings with a single fixed threshold, and that local validation is warranted before implementation. Nonetheless, a cutoff of < 2 retains utility as a rule‐out criterion given its high negative predictive value; threshold selection, however, should remain context‐specific rather than universally standardized.

Beyond the between‐hospital variability described earlier, the performance of the SADFUL scoring system in the original derivation study was notably higher than in our current external validation overall (AUROC 0.78, 95% CI 0.74–0.81; sensitivity 75.6%, specificity 67.5% at the ≥ 3 cutoff) [12]. Several factors may be responsible for this discrepancy in performance compared to the derivation cohort. First, the patient inclusion criteria differed. In the derivation study, patients underwent initial screening for infectious diseases via a comprehensive chart review; in contrast, our study enrolled patients who underwent specific laboratory tests, a process which is not always driven by a high suspicion of bacteremia—and occasionally made as a routine step prior to antibiotic administration [20]—resulting in a substantially larger but more heterogeneous cohort. Second, the outcomes in this study only included blood culture test results obtained in the ED, not those collected from the intensive care unit or general ward. This might account for the significantly lower positive rate of true bacteremia in the current study (2.2%) compared with that in the derivation cohort (11.0%). To further account for potential selection bias and confounding, we performed a sensitivity analysis using PSM, which balanced baseline characteristics and yielded a slightly lower AUROC (0.62). This confirms that a portion of the score’s predictive power in the unmatched cohort was attributable to confounding rather than the score itself.

The observed decrease in the SADFUL score’s performance upon external validation is a common phenomenon in prediction model research. This pattern of performance degradation has been reported for other bacteremia scores as well. For example, the external validation of the Shapiro rule showed a numerical drop in AUROC from 0.80 to 0.75, and the ID‐BactER score showed a similar drop from 0.80 to 0.74 (Supporting Table 2) [6, 21]. While the statistical significance of these specific declines was not formally reported in those studies, this consistent trend highlights the challenge of generalizability and the risk of overfitting in derivation models. Our findings for the SADFUL score align with these observations.

Given this context of modest performance, the score’s clinical utility must be carefully defined by metrics that are less dependent on prevalence. Our analysis of PLR and NLR clarifies this role. The PLR for the SADFUL score was consistently low (ranging from 1.33 to 2.37), confirming that it is ineffective as a confirmatory tool. However, its consistently high NPV (> 0.98) and moderately low NLR (ranging from 0.47 to 0.77) suggest its primary potential lies as a tool of exclusion. Accordingly, the SADFUL score should be positioned as a rule‐out aid rather than a conclusive diagnostic test for bacteremia, complementing—rather than replacing—clinical judgment and confirmatory blood culture results. In this role, the simplicity of the SADFUL score—which uses only six components—offers a practical advantage over more complex models, potentially helping clinicians identify low‐risk EDR patients who may not require blood cultures. That said, this study highlights that the score’s discriminative performance and optimal threshold vary meaningfully across institutional settings—a limitation that may stem from its current parameter design, differences in patient case mix, or omission of relevant variables. Taken together, these findings suggest that the SADFUL score is not yet suitable for universal implementation and that local validation should precede clinical adoption.

## 5. Limitations

This study had several important limitations. First, our findings are subject to two key sources of selection bias. The primary source is the requirement for blood cultures in the inclusion criteria. Because blood cultures are drawn only when emergency physicians already suspect a bloodstream infection, our cohort represents a preselected, higher acuity population rather than the broader, undifferentiated EDR population. In our dataset, 22,410 (78.4%) of 28,592 EDR patients lacked blood cultures and were therefore excluded, comprising 82.1% of EDR patients in Hospital A and 71.8% in Hospital B (Figure 1). Patients with viral infections or milder infectious conditions—for whom blood cultures were not ordered—were systematically excluded. This enrichment for clinical suspicion likely inflates the apparent sensitivity of the SADFUL score and limits the generalizability of our findings to all EDR patients, as the score has not been tested in the lower‐acuity majority of EDR patients in whom clinicians did not initially suspect bacteremia.

Second, by focusing exclusively on the EDR cohort, our findings cannot be generalized to the broader population of all ED patients with suspected bacteremia. Furthermore, this study focused strictly on the diagnostic performance of the SADFUL score; consequently, clinical outcomes such as hospital mortality were not evaluated as study endpoints.

Third, we observed a significant difference in blood culture utilization rates between the two hospitals, which introduces a potential source of unmeasured confounding (Supporting Table 1). While this difference could be an indicator of a sicker patient population in the rural regional hospital, it could equally reflect variations in local practice patterns or institutional protocols. Our retrospective design does not allow us to disentangle these factors, which may impact the direct comparison of the performance metrics for each hospital.

Fourth, the retrospective data collection, which relied on manual chart abstraction, may be subject to information bias from incomplete or unrecorded clinical data. Specifically, certain high‐risk comorbidities, such as liver cirrhosis, were not captured in our clinical dataset, which may limit the comprehensive evaluation of the score in certain vulnerable subpopulations. Finally, as the study was conducted in two hospitals in Taiwan, local practice patterns could influence the results. Future validation in different healthcare systems is warranted to establish broader generalizability.

## 6. Conclusion

Based on the external validation, the SADFUL score demonstrated fair to moderate discriminative ability for bacteremia in ED revisit patients, with performance and optimal cutoffs varying between the two hospital settings. The score should therefore be regarded as a tool to help rule out bacteremia in low‐risk EDR patients rather than as a conclusive diagnostic test, and local validation is crucial before clinical implementation to establish an appropriate, context‐specific cutoff. Future prospective, multicenter studies, including interventional trials assessing its impact on blood culture utilization and antibiotic stewardship, are warranted to develop a tool with both better generalizability and higher specificity.

## Author Contributions

Cheng‐Yi Fan, Chun‐Ju Lien, and Po‐Lin Wang acquired the data. Chi‐Hsin Chen and Jiun‐Wei Chen analyzed the data. Cheng‐Yi Fan interpreted the data and drafted the manuscript. Edward Pei‐Chuan Huang and Chih‐Wei Sung revised the manuscript. Chih‐Wei Sung designed and managed the study.

## Funding

This work was funded by the National Taiwan University Hospital Hsin‐Chu Branch (grant number 110‐HCH019, 115‐GH016 and 115‐GH018).

## Ethics Statement

This study was approved by the Institutional Review Board of the National Taiwan University (No. 202305135RINB). Informed consent was waived.

## Consent

Please see the Ethics Statement.

## Conflicts of Interest

The authors declare no conflicts of interest.

## Supporting Information

Additional supporting information can be found online in the Supporting Information section.

## Supporting information


**Supporting Information** Supporting Figure 1. The receiver operating characteristic curve of the SADFUL score in the sensitivity analysis. Supporting Figure 2. The flexible calibration curves using the SADFUL score for identifying bacteremia in the sensitivity analysis. Supporting Table 1. Basic information in the two hospitals. Supporting Table 2. Comparison of elements in different clinical decision rules. Supporting Table 3. Identified pathogens in true bacteremia. Supporting Table 4. Multiple logistic regressions on bacteremia. Supporting Table 5. Baseline characteristics in the bacteremia and matched nonbacteremia cohorts in the sensitivity analysis. Supporting Table 6. Performance of the SADFUL score with different cutoffs in the sensitivity analysis. This study was reported in accordance with the Strengthening the Reporting of Observational Studies in Epidemiology (STROBE) guidelines for cohort studies. The completed STROBE checklist is provided as Supporting Information (STROBE_checklist_R2_2).

## Data Availability

The data that support the findings of this study are available from the corresponding author upon reasonable request.
