# Supplementary material for: Geographic Validation of the SADFUL Scores for Identifying Bacteremia in the Unscheduled Emergency Department Revisit Cohorts
Source: Emerg Med Int. 2026 Jun 16;2026:1195292. doi: 10.1155/emmi/1195292 (PMC13270252; doi:10.1155/emmi/1195292)
Supplement: Supplementary file 1 — Supporting Information Supporting Figure 1. The receiver operating characteristic curve of the SADFUL score in the sensitivity analysis. Supporting Figure 2. The flexible calibration curves using the SADFUL score for identifying bacteremia in the sensitivity analysis. Supporting Table 1. Basic information in the two hospitals. Supporting Table 2. Comparison of elements in different clinical decision rules. Supporting Table 3. Identified pathogens in true bacteremia. Supporting Table 4. Multiple logistic regressions on bacteremia. Supporting Table 5. Baseline characteristics in the bacteremia and matched nonbacteremia cohorts in the sensitivity analysis. Supporting Table 6. Performance of the SADFUL score with different cutoffs in the sensitivity analysis. This study was reported in accordance with the Strengthening the Reporting of Observational Studies in Epidemiology (STROBE) guidelines for cohort studies. The completed STROBE checklist is provided as Supporting Information (STROBE_checklist_R2_2). [file EMMI-2026-1195292-s001.zip › Supplementary Tables_R2.docx]

| Supplemental Table 1. Basic information in the two hospitals | | |
| --- | --- | --- |
|  | Hospital A | Hospital B |
| Hospital size | Academic medical center | Rural district-level hospital |
| Hospital name | National Taiwan University Hospital | National Taiwan University Hospital Yun-Lin Branch |
| Location | Taipei City | Yun-Lin County |
| ED visit/month | 9,000 | 4,000 |
| Blood culture/month | 2500 patients | 1800 patients |
| Blood culture/ED visit | 0.28 | 0.45 |
| Blood culture vial | BD BACTEC Plus Aerobic/Anaerobic Culture Vials | |
| General ward beds | More than 2,500 | 700-800 |
| ED: emergency department | | |

| Supplemental Table 2. Comparison of elements in different clinical decision rules | | | | | | | |
| --- | --- | --- | --- | --- | --- | --- | --- |
| Clinical Parameter | SADFUL | SIRS | qSOFA | Shock Index | Shapiro Rule  (Major) | Shapiro Rule (Minor) | ID-BactER Score |
| Age (years) | > 55 (+1 pt) |  |  |  |  | > 65 (1 pt) | > 65 (1 pt) |
| Diabetes mellitus | Yes (+1 pt) |  |  |  |  |  |  |
| Symptoms | URI symptoms  (-2 pts) |  |  |  |  | Chills (1 pt)  Vomiting  (1 pt) | Chills (2 pts)  Vomiting  (1 pt) |
| Clinical Suspicion |  |  |  |  | Suspect IE  (3 pts)  Indwelling vascular catheter (2 pts) |  |  |
| Vital Signs |  |  |  |  |  |  |  |
| Temperature (°C) | ≥ 38 (+2 pts) | < 36 or ≥ 38 |  |  | > 39.4 (3 pts) | 38.3–39.3  (1 pt) | ≥ 38.0 (1 pt) |
| Heart Rate (bpm) |  | > 90 |  | HR/SBP |  |  |  |
| Respiratory Rate (/min) |  | > 20 | ≥ 22 |  |  |  |  |
| Systolic BP (mmHg) |  |  | ≤ 100 | HR/SBP |  | < 90 (1 pt) | < 90 (1 pt) |
| Mental Status (GCS) |  |  | < 15 |  |  |  | Altered mental status (1 pt) |
| Physical Examination |  |  |  |  |  |  |  |
| Abdominal Exam |  |  |  |  |  |  | Focal abdominal sign (1 pt) |
| Laboratory Data |  |  |  |  |  |  |  |
| WBC (K/μL) | < 4 (+2 pts) | < 4 or > 12 |  |  |  | > 18 (1 pt) | > 15 (1 pt) |
| Differential Count | Segmented neutrophils > 85% (+3 pts) |  |  |  |  | Bands > 5%  (1 pt) |  |
| Platelets (/mm³) |  |  |  |  |  | < 150K (1 pt) | < 150K (1 pt) |
| BUN (mg/dL) |  |  |  |  |  |  | > 20 (1 pt) |
| Creatinine (mg/dL) |  |  |  |  |  | > 2.0 (1 pt) |  |
| CRP (mg/dL) |  |  |  |  |  |  | > 10 (1 pt) |
| Decision Threshold | Sum of points | ≥ 2 criteria | ≥ 2 criteria | Ratio ≥ 1 | ≥ 1 major criterion | ≥ 2 minor criteria | Sum of points |
| BUN: blood urea nitrogen; CRP: C-reactive protein; GCS: Glasgow Coma Scale; IE: infective endocarditis; URI: upper respiratory tract infection; WBC: white blood cell; | | | | | | | |

| Supplemental Table 3. Identified pathogens in true bacteremia | | |
| --- | --- | --- |
| Pathogen | Count | Rate (%) |
| Staphylococcus aureus | 28 | 22.6 |
| Pseudomonas aeruginosa | 13 | 10.5 |
| Salmonella O9(group D1 ) | 7 | 5.7 |
| Staphylococcus epidermidis | 7 | 5.7 |
| Streptococcus agalactiae | 6 | 4.9 |
| Streptococcus anginosus | 6 | 4.9 |
| Serratia marcescens | 5 | 4.1 |
| Staphylococcus hominis | 4 | 3.2 |
| Parabacteroides distasonis | 3 | 2.4 |
| Proteus mirabilis | 3 | 2.4 |
| Staphylococcus capitis | 3 | 2.4 |
| Staphylococcus warneri | 3 | 2.4 |
| Streptococcus dysgalactiae | 3 | 2.4 |
| Plesiomonas shigelloides | 2 | 1.6 |
| Prevotella bivia | 2 | 1.6 |
| Staphylococcus haemolyticus | 2 | 1.6 |
| Streptococcus constellatus | 2 | 1.6 |
| Streptococcus gordonii | 2 | 1.6 |
| Streptococcus vestibularis | 2 | 1.6 |
| Nonfermentative GNB | 1 | 0.8 |
| Paenibacillus sp | 1 | 0.8 |
| Parvimonas micra | 1 | 0.8 |
| Prevotella intermedia | 1 | 0.8 |
| Prevotella species | 1 | 0.8 |
| Providencia rettgeri | 1 | 0.8 |
| Pseudomonas alcaligenes | 1 | 0.8 |
| Pseudomonas otitidis | 1 | 0.8 |

| Supplemental Table 4. Multiple logistic regressions on bacteremia | | | | |
| --- | --- | --- | --- | --- |
| Hospital | Variables | aOR | 95% CI | *p* |
| Hospital  A | Age (year) | 1.01 | 0.99 - 1.02 | 0.474 |
|  | Hypertension | 1.75 | 1.02 – 3.00 | 0.042 |
|  | CAD | 1.34 | 0.74 - 2.42 | 0.332 |
|  | CKD | 2.19 | 1.28 - 3.77 | 0.004 |
|  | Chills | 1.36 | 0.78 - 2.37 | 0.283 |
|  | Body temperature |  |  |  |
|  | 36℃ - 38℃ | Ref. | | |
|  | <36℃ | 1.41 | 0.49 - 4.03 | 0.525 |
|  | >38℃ | 1.83 | 1.07 - 3.12 | 0.026 |
|  | Segmented neutrophils (%) | 1.01 | 0.99 - 1.03 | 0.205 |
| Hospital  B | Age | 1.01 | 1.00 - 1.03 | 0.117 |
|  | CKD | 1.43 | 0.71 - 2.91 | 0.316 |
|  | Chills | 1.57 | 0.69 - 3.58 | 0.287 |
|  | Triage level 1 or 2 | 1.26 | 0.61 - 2.6 | 0.528 |
|  | Body temperature |  |  |  |
|  | 36℃ - 38℃ | Ref. | | |
|  | <36℃ | 1.28 | 0.37 - 4.45 | 0.697 |
|  | >38℃ | 3.87 | 2.04 - 7.32 | <0.001 |
|  | Breath rate > 20 time/min | 3.41 | 1.47 - 7.91 | 0.004 |
|  | SBP < 100 mmHg | 3.59 | 1.54 - 8.34 | 0.003 |
|  | WBC |  |  |  |
|  | 4 - 12 K/μL | Ref. | | |
|  | <4 K/μL | 2.70 | 1.04 - 7.03 | 0.042 |
|  | >12 K/μL | 1.45 | 0.76 - 2.75 | 0.257 |
| aOR=adjusted odds ratio, CI=confidence interval, CAD: coronary artery disease; CKD: chronic kidney disease; SBP: systolic blood pressure; WBC: white blood cell | | | | |

| Supplemental Table 5. Baseline characteristics in the bacteremia and matched non-bacteremia cohorts in the sensitivity analysis | | | |
| --- | --- | --- | --- |
| Dataset | Bacteremia  (n=120), n(%) | Non-bacteremia  (n=480), n(%) | *p*-value |
| Age (year) | 67.2±18.0 | 67.5±17.1 | 0.845 |
| >55 years old | 93(77.5) | 378(78.8) | 0.766 |
| Males | 68(56.7) | 290(60.4) | 0.454 |
| Pre-existing diseases |  |  |  |
| Hypertension | 62(51.7) | 250(52.1) | 0.935 |
| DM | 39(32.5) | 155(32.3) | 0.965 |
| CAD | 24(20.0) | 107(22.3) | 0.587 |
| CVA | 9(7.5) | 50(10.4) | 0.337 |
| Cancer | 41(34.2) | 172(35.8) | 0.733 |
| CKD | 34(28.3) | 127(26.5) | 0.678 |
| Chief complaints |  |  |  |
| Upper respiratory tract | 19(15.8) | 93(19.4) | 0.373 |
| Gastrointestinal tract | 56(46.7) | 223(46.5) | 0.967 |
| Urinary tract | 10(8.3) | 74(15.4) | 0.045 |
| Dyspnea | 22(18.3) | 93(19.4) | 0.795 |
| Chills | 28(23.3) | 109(22.7) | 0.884 |
| Triage level 1 or 2 | 38(31.7) | 145(30.2) | 0.756 |
| Vital signs |  |  |  |
| GCS<15 | 13(10.8) | 56(11.7) | 0.798 |
| Body temperature |  |  | 0.001 |
| <36℃ | 7(5.8) | 33(6.9) |  |
| >38℃ | 51(42.5) | 120(25.0) |  |
| Heart rate > 90 bpm | 77(64.2) | 264(55.0) | 0.070 |
| Breath rate > 20 time/min | 21(17.5) | 68(14.2) | 0.358 |
| SBP < 100 mmHg | 21(17.5) | 91(19.0) | 0.714 |
| Laboratory Test |  |  |  |
| WBC |  |  | 0.018 |
| <4 K/μL | 13(10.8) | 35(7.3) |  |
| >12 K/μL | 42(35.0) | 119(24.8) |  |
| Segmented neutrophils (%) | 79.2±17.6 | 76.8±13.9 | 0.111 |
| Admission | 102(85.0) | 305(63.5) | <0.001 |
| Length of stays | 16.0 [10.0, 30.0] | 10.0 [6.0, 16.0] | <0.001 |
| CAD: coronary artery disease; CKD: chronic kidney disease; CT: computed tomography; CVA: cerebrovascular accident; DM: diabetes mellitus; GCS: Glasgow coma scale; SBP: systolic blood pressure; WBC: white blood cell | | | |

| Supplemental Table 6. Performance of the SADFUL score with different cut-offs in the sensitivity analysis | | | | | | | | | |
| --- | --- | --- | --- | --- | --- | --- | --- | --- | --- |
|  | SADFUL | Sensitivity | Specificity | PPV | NPV | PLR | NLR | DOR | Accuracy |
| PSM cohort | ≥2 | 0.77 | 0.43 | 0.25 | 0.88 | 1.33 | 0.55 | 2.43 | 0.49 |
|  | ≥3 | 0.56 | 0.59 | 0.25 | 0.84 | 1.36 | 0.75 | 1.82 | 0.58 |
|  | ≥4 | 0.48 | 0.69 | 0.28 | 0.84 | 1.52 | 0.76 | 1.99 | 0.65 |
| DOR: diagnostic odds ratio; NLR: negative likelihood ratio; NPV: negative predictive value; PLR: positive likelihood ratio; PPV: positive predictive value; PSM: propensity score matching | | | | | | | | | |
