# Supplementary figures and images for: Geographic Validation of the SADFUL Scores for Identifying Bacteremia in the Unscheduled Emergency Department Revisit Cohorts
Source: Emerg Med Int. 2026 Jun 16;2026:1195292. doi: 10.1155/emmi/1195292 (PMC13270252; doi:10.1155/emmi/1195292)

## ROC Curve

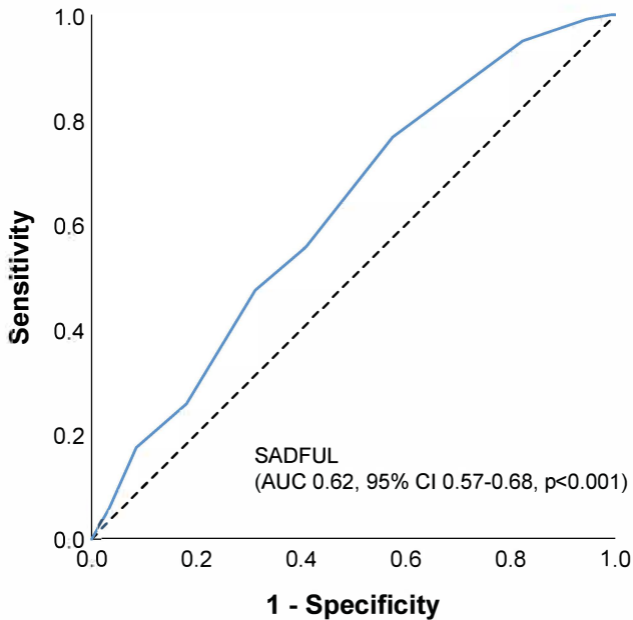

Supplement: Supplementary file 1 — Supporting Information Supporting Figure 1. The receiver operating characteristic curve of the SADFUL score in the sensitivity analysis. Supporting Figure 2. The flexible calibration curves using the SADFUL score for identifying bacteremia in the sensitivity analysis. Supporting Table 1. Basic information in the two hospitals. Supporting Table 2. Comparison of elements in different clinical decision rules. Supporting Table 3. Identified pathogens in true bacteremia. Supporting Table 4. Multiple logistic regressions on bacteremia. Supporting Table 5. Baseline characteristics in the bacteremia and matched nonbacteremia cohorts in the sensitivity analysis. Supporting Table 6. Performance of the SADFUL score with different cutoffs in the sensitivity analysis. This study was reported in accordance with the Strengthening the Reporting of Observational Studies in Epidemiology (STROBE) guidelines for cohort studies. The completed STROBE checklist is provided as Supporting Information (STROBE_checklist_R2_2). [file EMMI-2026-1195292-s001.zip › S.Figure 1.pdf]

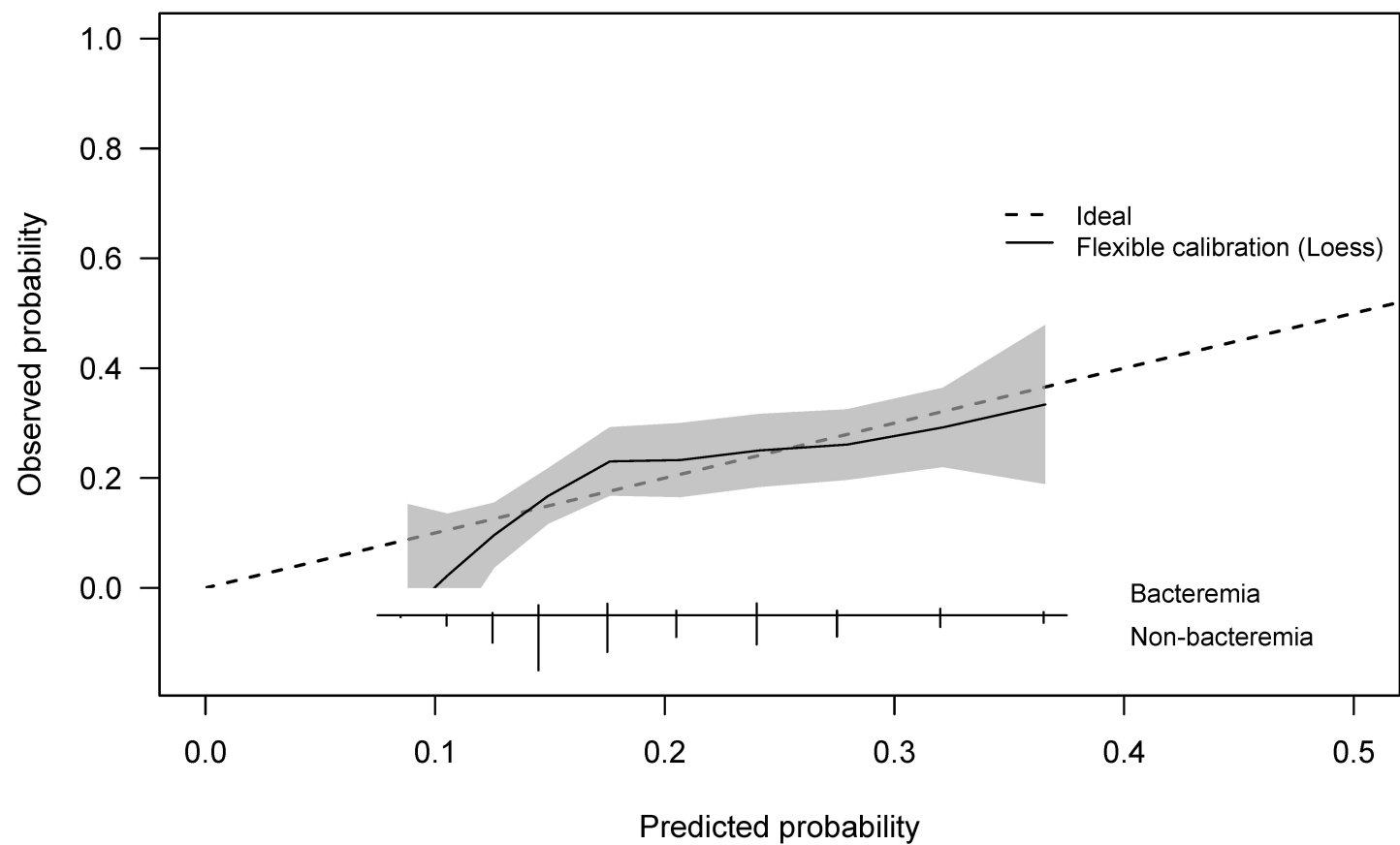

Supplement: Supplementary file 1 — Supporting Information Supporting Figure 1. The receiver operating characteristic curve of the SADFUL score in the sensitivity analysis. Supporting Figure 2. The flexible calibration curves using the SADFUL score for identifying bacteremia in the sensitivity analysis. Supporting Table 1. Basic information in the two hospitals. Supporting Table 2. Comparison of elements in different clinical decision rules. Supporting Table 3. Identified pathogens in true bacteremia. Supporting Table 4. Multiple logistic regressions on bacteremia. Supporting Table 5. Baseline characteristics in the bacteremia and matched nonbacteremia cohorts in the sensitivity analysis. Supporting Table 6. Performance of the SADFUL score with different cutoffs in the sensitivity analysis. This study was reported in accordance with the Strengthening the Reporting of Observational Studies in Epidemiology (STROBE) guidelines for cohort studies. The completed STROBE checklist is provided as Supporting Information (STROBE_checklist_R2_2). [file EMMI-2026-1195292-s001.zip › S.Figure 2.pdf]
